# SURGICAL SITE INFECTION IN RESECTIONS OF DIGESTIVE SYSTEM TUMOURS

**DOI:** 10.1590/0102-6720202400024e1817

**Published:** 2024-08-19

**Authors:** Adriano Carneiro da Costa, Fernando Santa-Cruz, Ana Valéria Torres, Eduarda Augusta de Lucena Caldas, Alessandro Mazzota, Flávio Kreimer, Álvaro Antônio Bandeira Ferraz

**Affiliations:** 1 Universidade Federal de Pernambuco, Hospital Universitário, Department of Surgery – Recife (PE), Brazil; 2 Institute Mutualiste Montsouris, Metabolic and Oncological Surgery, Department of Digestive – Paris, France.

**Keywords:** Surgical Wound Infection, Digestive System, Neoplasms, Surgical Oncology, Infecção da Ferida Cirúrgica, Sistema Digestório, Neoplasias, Oncologia Cirúrgica

## Abstract

Postoperative infectious complications are extremely important to surgeons and the entire medical care team. Among these complications, surgical site infection (SSI) appears to be one of the earliest and most prevalent events and is considered an inherent complication of surgical procedures. In oncological patients submitted to resections of digestive system tumors, there is a confluence of several risk factors for SSI, making it necessary to establish measures to maximize the control of this condition to provide a better prognosis for these patients. Some risk factors for SSI are the manipulation of structures hosting the highest density of pathogenic microorganisms, such as the colon, the patient's performance status, the patient's nutritional status, the use of chemotherapy and/or radiotherapy, and the surgical procedure itself, which tends to last longer and be more complex than surgeries for benign conditions of the digestive system. Therefore, this review sought to provide a qualitative analysis and a summary of the literature regarding the SSI of postoperative tumor patients who underwent surgical resection and were well-structured postoperatively, to provide objective data on this problem, and alert about the well-structured needs of individualized pre-, peri-, and post-protocols to avoid the development of these events.

## RESUMO

As complicações pós-operatórias de origem infecciosa são de extrema importância para o cirurgião e toda a equipe de assistência médico-hospitalar. Dentre essas complicações, a infecção de sítio cirúrgico (ISC) se configura como um dos eventos mais precoces e mais prevalentes, sendo considerada uma complicação intrínseca ao procedimento. Em pacientes oncológicos submetidos a ressecções de tumores do aparelho digestivo, existe uma confluência de diversos fatores de risco para ISC, tornando-se necessário o estabelecimento de medidas para controlar ao máximo essa condição, e, assim, proporcionar um melhor prognóstico a esses pacientes. O manuseio de estruturas com elevada densidade de microrganismos patogênicos, como o cólon, o *performance status* do paciente, o estado nutricional, a realização de quimioterapia e/ou radioterapia, além do próprio procedimento cirúrgico em si, em geral prolongado e mais complexo quando comparado com patologias benignas deste sistema, são alguns dos fatores de risco que contribuem para um aumento na incidência de ISC nessa população. Assim, esta revisão buscou fornecer uma análise qualitativa e um resumo da literatura publicada acerca da ISC nos pacientes submetidos a ressecções de tumores malignos do aparelho digestivo e bem estruturados no pós-operatório, visando obter dados objetivos do problema e alertar sobre a necessidade de protocolos pré-, peri- e pós-operatórios bem-estruturados e individualizados, capazes de prevenir a ocorrência desses eventos.

## INTRODUCTION

Surgical site infection (SSI) is the most common nosocomial infection in surgical patients, with an incidence of 10–30% in digestive system procedures^12-14,25^. It is one of the causes of morbidity, longer hospital stays, and readmission, lowering the quality of life of patients and generating expenses of up to 10 billion dollars annually in the USA^17,37^. SSI is consistently reported to be responsible for up to 25% of all healthcare-associated infections and is the most prevalent complication arising in major gastrointestinal surgeries^1,7,11^. It is common in such surgeries because these manipulate areas with a high density of pathogenic microorganisms, so measures are needed to control the risk of postoperative complications as much as possible. Among surgeries of the digestive system, those for neoplasia, especially hepatectomy, subtotal esophagectomy, and pancreatoduodenectomy, have the highest rates of postoperative complications, especially infection^7,9,16,30,37^.

Furthermore, surgeries for the resection of bile duct tumors, especially extrahepatic cholangiocarcinoma, bring a high risk of postoperative complications, such as anastomotic leakage and SSI, with deep space infection^4,8^. Colorectal resection surgeries also have a high risk due to the involvement of contaminated lumen^15,26^. Even with adequate preoperative antibiotic measures and aseptic techniques, SSI has a prevalence rate between 15 and 30%^28^.

In this study, a literature review was conducted on SSI and its control measures in patients with digestive tract cancer.

## METHODS

This systematic review followed the Preferred Reporting Items for Systematic Review (PRISMA) 2020 guidelines.

### Literature searches

A literature review was performed to evaluate the currently available information on SSIs in oncological resections of the digestive tract. Articles published in English between 2007 and 2019 were accepted. The search, using an algorithm prospectively defined in PubMed, was performed on March 10, 2019. Data were obtained from the National Library of Medicine (PubMed) and the Scientific Electronic Library Online (SciELO) databases. The keywords used were "surgical site infection", "antibiotic prophylaxis", "surgical oncology", and "infection control and digestive system surgical procedures". These terms were combined using Boolean operators ("AND", "OR") to refine the research.

The first search for "surgical site infection and surgical oncology" yielded 914 articles. After excluding duplicates and screening the abstracts for relevance, 71 articles were selected.

In the second search, the terms "surgical site infection" and "antibiotic prophylaxis and digestive system surgical procedures" were used, yielding 118 studies. After screening the abstracts, 58 articles were considered.

A total of 129 full-text articles were evaluated for eligibility. Of these, 35 were selected by applying the eligibility criteria of the present study.

### Inclusion and exclusion criteria

The inclusion criteria were as follows: articles published in the last five years with full text available and conducted on humans. Experimental and review articles involving animal models were excluded.

### Data extraction

Two authors independently extracted the data, and the senior authors resolved any disagreements after discussion.

## RESULTS

**Figure 1:**
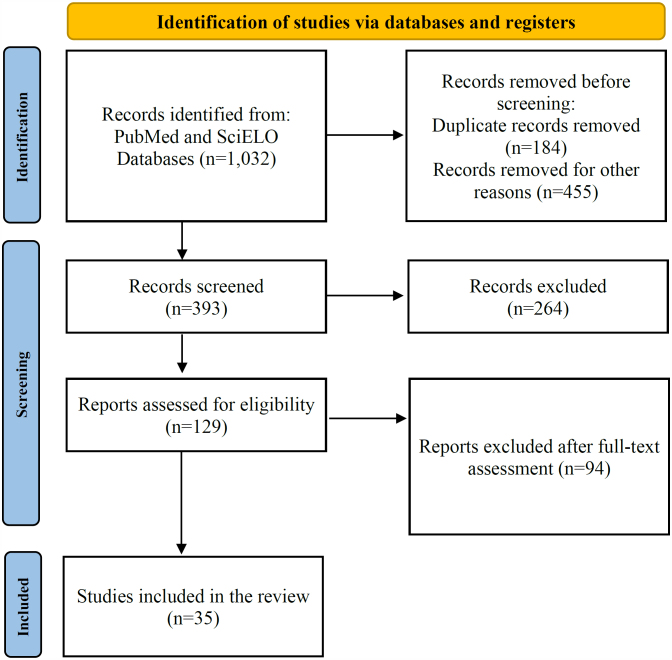
Studies included (PRISMA).

The literature search yielded 1,032 references. After duplicate records and screened titles and abstracts were removed, 129 articles met all the selection criteria. The reference search of the included studies yielded no additional studies suitable for inclusion. A total of 94 articles were excluded due to study characteristics or methods, leaving 35 articles for analysis (Figure 1).

## DISCUSSION

### Surgical site infection

#### General aspects

SSI is defined as an infection that occurs between 30 and 90 days after a surgical procedure and affects the incision area and/or deep tissues at the surgical site. The infection may be superficial or deep incisional or involve organs or visceral spaces^5^.

Incisional SSI is associated with purulent secretion with a positive culture^30^. Perioperative wound management is a determining factor in the occurrence of SSI and includes the use of antibiotic prophylaxis, skin preparation, maintenance of normothermia, and subcutaneous lavage before the incision is closed, as well as suture care and skin dressings^19^.

### Risk factors

The risk factors for SSI may be related to the patient, the surgical procedure performed, and the pathogen involved. Thus, the 2017 Centers for Disease Control guidelines recommend the use of specific interventions for preventing SSI, such as antibiotic prophylaxis, glycemic control, temperature control (perioperative maintained normothermia), oxygenation, and antisepsis of the operative field immediately before the incision. This evidence has a high level of recommendation, but it depends on the conditions and peculiarities of each patient and the surgery to be performed^5,6^.

Perioperative hyperglycemia is associated with postoperative surgical complications and a higher risk of SSI^23,24^. Ata et al. evaluated postoperative hyperglycemia in 226 colorectal surgery cases and concluded that blood glucose above 140 mg/dL favored the development of SSI^2^. In a study by Guzman-Pruneda et al., an association of some conditions with a reduced risk of SSI was observed, such as nonsmoker status, minimally invasive surgery, and compliance with five measures preestablished for the method applied, such as the use of chlorhexidine wipes for cleaning the skin on the day of surgery, bathing the night before the procedure, the use of antibiotics intravenously and at the time of anesthetic induction, skin preparation with ChloraPrep™, appropriate shaving, and, only when necessary, mechanical bowel preparation^15^. Neumayer et al. reported an increase in SSI under the following preoperative conditions: diabetes, alcoholism, steroid use, recent radiotherapy, preoperative low albumin, gastrointestinal tract surgery, and emergency surgery^27^. Obesity, nutritional status, and prolonged preoperative hospitalization were also important risk factors for SSI^29^.

Regarding the surgical procedure, there are two main parameters to be evaluated: the duration of the operation and the surgical technique. Depending on these conditions, difficulties with hemostasis, failure of dead space obliteration, and surgical trauma may occur^18^.

### The cancer patient

Regarding gastrointestinal malignancies, patients who undergo elective surgery for cancer are at high risk of developing postoperative complications. Such conditions result from factors including malnutrition, diet, and stress associated with the surgery. An example is patients who undergo esophagectomy. These patients are occasionally malnourished and usually have impaired immune function due to difficulty swallowing and anorexia related to preoperative treatment^21,31^.

Nutritional support is the most important option for reducing the incidence of infectious and noninfectious postoperative complications, in addition to enhancing patient's immunity and, eventually, decreasing hospitalization time, and healthcare costs. Nutritional supplementation may also improve the prognosis of the patient^34^. The current practice of reintroducing oral nutrition as early as possible in postoperative patients is well-established for several situations. Some studies have shown that this practice is feasible and safe after upper gastrointestinal surgery and may reduce infection-related potential complications and hospitalization time compared to the traditional zero-diet approach^25,31^.

Postoperative infection predicts clinical adverse outcomes in patients with malignancies, such as colorectal cancer. In these cases, the incidence of SSI is 5–30%, and SSI is more common in patients who undergo rectal surgery than in those who undergo colon surgery. In colorectal cancer, perioperative mortality after elective colorectal surgery is 3–4%^22^.

High morbidity, high mortality, and compromised long-term cancer outcomes have been reported after deep SSI in rectal tumor resections^37^. Regarding colorectal tumors, after chemotherapy/radiotherapy, deep SSIs and dehiscence are more prevalent. It is possible that this is due to a stimulus to inflammation and fibrosis, in addition to impairment of microcirculation in the pelvic area around the tumor, which predisposes patients to higher vulnerability to infection. Furthermore, local tissue toxicity and systemic effects caused by chemotherapy and radiotherapy impair wound healing^36^.

Patients with other types of malignancies who undergo oncological resection procedures include those with hepatocellular carcinoma. The SSI morbidity rate associated with hepatectomy has decreased with improvements in surgical techniques and perioperative management. Nevertheless, there is still a high incidence of postoperative infections, ranging from 15.6 to 25%^33^. SSI has also been observed, especially in patients with liver-related comorbidities such as cirrhosis^32^.

Surgery, usually gastroduodenopancreatectomy (Whipple surgery) or pylorus-sparing pancreatoduodenectomy, is the only curative treatment available for cancer of the head of the pancreas. Because it is highly invasive, this surgery is associated with serious complications that considerably reduce survival, such as pancreatic fistula, gastrojejunostomy escape, and SSI^7^. Preoperative serum amylase, glucose, creatinine, albumin, bilirubin, aspartate aminotransferase, alanine aminotransferase, alkaline phosphatase, white blood cell count, hematocrit, and platelet count measured within two weeks of surgery are considered predictors of morbidity after pancreatoduodenectomy^27^.

In surgeries for metastatic cancer, Kamboj et al. found, in addition to a higher risk of SSI in general, a higher frequency of infections of the visceral spaces compared to other types of non-oncological surgeries and those for localized tumors^18^.

Major oncological resections are associated with high morbidity and even mortality risk, with SSI being one of the most common complications^13^. Furthermore, surgical complications such as SSI delay the initiation of chemotherapy and radiotherapy in these patients, which may significantly shorten their survival^3,19^.

Thus, given all these variables, there are global guidelines that advocate the use of intensive pre- and perioperative protocols for SSI control in patients undergoing surgical procedures of the digestive tract, especially those for malignant neoplasms^20,35^.

### Infection control in surgery

In Brazil, SSI is one of the main infectious complications in healthcare settings, ranking third among all infections in healthcare facilities and accounting for 14–16% of infections in hospitalized patients. Infected patients are twice as likely to die or spend some time in the intensive care unit, in addition to being five times more likely to be readmitted after discharge^32^.

Around 30% of nosocomial infections are preventable, and from this perspective^14^, Ferraz et al., as part of a committee for nosocomial infection control, reported on the importance of certain practices for the control of SSI, such as accurate diagnosis of infections, body hygiene, control of associated conditions, minimal preoperative hospitalization, trichotomy care, antisepsis and rigorous asepsis, adequate and delicate surgical techniques, dissemination of the results of the committee and the infection/surgeon/anesthetist ratio, and strict control of the prescription of antimicrobials^12,17^. Additionally, hand hygiene measures, appropriate wound care, infection prevention devices, and targeted detection and decolonization of patients infected with methicillin-resistant *Staphylococcus aureus* (MRSA) are measures that can reduce MRSA infection worldwide^33^.

### Antibiotic prophylaxis

Prophylaxis is associated with the prevention of infection and can be primary, secondary, or eradicated. The primary objective is to prevent the development of an infection, whereas the secondary objective is to avoid the recurrence or reactivation of a preexisting infection. As for eradication, this approach aims to eliminate colonies of microorganisms that may cause an infection, thus preventing any later infections^12,35,37^.

Interventions to decrease the risk of known SSIs include antibiotic prophylaxis, oxygen supply, fluid control, bowel preparation, and skin disinfection. Antibiotic prophylaxis reduces the risk of SSI, and its principles date back to 1950^6^. Four points are required for optimal coverage: time of onset, choice of the right antibiotic, and the dose and duration of the antibiotic. The duration of treatment that is best for preventing SSI is still unknown^14,36^.

The most common agent causing superficial SSI is *Staphylococcus aureus*. The most common causative agents in gastroduodenal procedures are coliform bacteria (*Escherichia coli, Proteus* species, *Klebsiella* species), *Staphylococcus, Streptococcus, Enterococcus*, and occasionally *Bacteroides* ^35^.

The use of one dose of cefazolin, with a high level of evidence, is recommended for procedures involving opening the lumen of the intestinal tract^35^. Studies have reported the use of antibiotics before surgical incisions, with an additional dose depending on the half-life of the antimicrobial or according to the amount of fluid lost and administered during the surgical process^1,15,29^. In the literature, a better concentration of serum and tissue cephalosporin is reported in the surgical incision and at the end of the surgical procedure when this drug is administered during anesthetic induction^35^. Currently, the most common form of cefazolin used is as a bolus during the induction of anesthesia^37^.

With the popularization of laparoscopic surgery, there are still debates about the need for antibiotic prophylaxis in laparoscopic surgery patients. A recent study reported a benefit in the administration of antibiotic prophylaxis at the time of anesthesia induction for laparoscopic cholecystectomy, showing a balance between the prevention of infectious complications and the inadvertent use of antibiotics^8,35^. In recent studies, in patients undergoing open pancreatoduodenectomy, the use of piperacillin-tazobactam as perioperative prophylaxis reduced postoperative SSI, pancreatic fistula, and multiple sequelae of postoperative SSI. The results of these studies support the use of piperacillin-tazobactam as a standard treatment for open pancreatoduodenectomy^3,7,10^ (Table 1).

**Table 1:** Recommendations for antibiotic prophylaxis for the classes of germs most involved in digestive tract surgery^6,10^.

	Common pathogen	Recommended antibiotic
Stomach and small intestine	Gram-negative bacilli, Gram-positive cocci	Cefazolin
Bile ducts	Gram-negative bacilli, *Escherichia coli, Streptococcus*	Cefazolin, cefoxitin, or ampicillin/sulbactam
Liver	Gram-negative bacilli, *Streptococcus, Enterococci*	Cefazolin or ampicillin/sulbactam
Pancreas	Gram-positive	Ampicillin/sulbactam or Piperacillin-tazobactam
Colon/rectum	Gram-negative, anaerobic bacilli, *Enterococcus*	Cefazolin + metronidazole or cefoxitin or ampicillin/sulbactam

## CONCLUSIONS

It is important to establish pre-, peri-, and postoperative protocols for infection control in cancer patients undergoing resection of tumors of the digestive tract. Key measures, with a high level of evidence, such as the correct use of prophylactic antibiotics, glycemic control at acceptable levels, appropriate shaving, and preservation of nutritional status, along with secondary measures that theoretically reduce the risks of infection, such as prenatal baths, are needed. Surgery with chlorhexidine, loss of excess weight in the preoperative period, and maintenance of normothermia in the peri- and postoperative periods should be considered and applied systematically to get the best possible results.
